# Extracellular polymeric substances, a key element in understanding biofilm phenotype

**DOI:** 10.3934/microbiol.2018.2.274

**Published:** 2018-03-30

**Authors:** Patrick Di Martino

**Affiliations:** Groupe Biofilm et Comportement Microbien aux Interfaces, Laboratoire ERRMECe-EA1391, Université de Cergy-Pontoise, rue Descartes site de Neuville-sur-Oise 95031 Cergy-Pontoise, cedex France

**Keywords:** biofilm, matrix, extracellular polymeric substance, EPS, polysaccharide, amyloid, eDNA

## Abstract

One of the key elements in the establishment and maintenance of the biofilm structure and properties is the extracellular matrix. The extracellular matrix is composed of water and extracellular polymeric substances (EPS): primarily polysaccharides, proteins and DNA. Characterization of the matrix requires component identification, as well as determination of the relative concentration of EPS constituents, including their physicochemical properties and descriptions of their interactions. Several types of experimental approaches with varying degrees of destructiveness can be utilized for this characterization. The analysis of biofilm by infrared spectroscopy gives information about the chemical content of the matrix and the proportions of different EPS. The sensitivity of a biofilm to hydrolytic enzymes targeting different EPS gives insight into the composition of the matrix and the involvement of matrix components in the integrity of the structure. Using both chemical and physical treatments, extraction and purification of EPS from the biofilm also provides a means of determining matrix composition. Purified and/or artificial EPS can be used to obtain artificial matrices and to study their properties. Using examples from the literature, this review will illustrate selected technologies useful in the study of EPS that provide a better understanding of the structure-function relationships in extracellular matrix, and thus the structure-function relationships of the biofilm phenotype.

## 1. Introduction

A biofilm is a community of aggregated cells, organized as microcolonies living at an interface between a surface and a liquid phase and embedded in an organic polymer matrix of microbial origin. Bacteria-bacteria, bacteria-extracellular polymer, and bacteria-surface interactions drive the formation and behavior of a biofilm. Extracellular polymeric substances (EPS) are organic polymers of microbial origin involved in bacterial cells' interactions with their environment [1]. EPS are comprised of polysaccharides, proteins, extracellular DNA (eDNA), and lipids. Within biofilms, EPS are distributed between cells in a non-homogeneous pattern [2]. EPS interact with each other and form the matrix that encompasses microbial cells [3]. The stability of the matrix is ensured by non-covalent bonding between EPS that involves weak physicochemical forces. The EPS network interacting with bacterial aggregates confers cohesion and viscoelasticity to the structure [4]. The role of the different classes of EPS in biofilm behavior can be studied through various experimental approaches. Vibrational spectroscopy, like infrared spectroscopy, gives information about the chemical content of the biofilm and the different proportions of polysaccharides, proteins, and other EPS in the matrix. The use of hydrolytic enzymes targeting different EPS to destabilize biofilms provides information about the composition and the involvement of different matrix components in the integrity of the structure. The composition of the matrix can be determined by extraction and purification of EPS from the biofilm through physical and/or chemical treatments. In combination with bacterial cells, purified and/or artificial EPS can be used to obtain artificial biofilms and study their properties.

## 2. Analysis of the composition of biofilms by Fourier transform infrared spectroscopy

Infrared radiation corresponds to the electromagnetic spectrum from 0.78 to 1,000 μm. Fourier transform infrared (FT-IR) spectroscopy is an easily implemented technique that allows for the detection and identification of organic molecules and the study of microbial attachment and biofilm development [5],[6]. In the attenuated total reflection Fourier transform infrared (ATR/FT-IR) mode, the multiple reflections of the IR radiation on the inner surface of an internal reflection element (IRE) induces an evanescent wave of radiation that penetrates the adjacent environment to a depth of approximately 2 µm. Organic molecules near the outer surface of the IRE absorb the evanescent wave, resulting in molecular vibrations, which in turn cause in stretching and bending in chemical bond. The functional groups of the molecules absorb energy at specific wavelengths, which can be shifted in intensity or position by adjacent atoms. Thus, the resulting absorption spectrum is specific to the molecular composition of the sample. Biopolymers, bacterial cells, and biofilms can be analyzed by ATR/FT-IR. Band assignments of principal infrared vibration signals of the EPS of a biofilm are indicated in Table 1.

**Table 1.** Assignments of principal infrared vibrational signals of the 900–3000 cm^−1^ region of the ATR/FT-IR spectrum of a biofilm.

Windows of the IR spectra corresponding to EPS signals	Principal EPS	Main functional groups in biomolecules
2800–3000 cm^−1^	Lipids	CH, CH_2_, CH_3_
1500–1800 cm^−1^	Proteins	C=O, N-H, C-N (amide I, amide II)
900–1250 cm^−1^	Polysaccharides, nucleic acids	C-O, C-O-C, P=O, C-N, N-H (amide III)

When a mixture of the various biopolymers found in bacterial cells exists in the same proportions as that found in a cell, the IR spectrum has the principal characteristics of the spectrum of a living bacterium [6]. ATR/FT-IR spectra of living *Caulobacter crescentus* cells attached to a germanium crystal in high purity water show absorption bands at 1648, 1550, and 1306 cm^−1^ (corresponding to amide I, amide II, and amide III vibrational modes) and bands at 1454, 1397, 1341, 1246 and 1080 cm^−1^ assigned to the C-H bend, C-O stretch, the P=O stretch or amide III, and the C-O stretch (alcohols and carbohydrates) vibrational modes, respectively [5]. The ATR/FT-IR spectra of *Pseudomonas fluorescens* planktonic cells harvested at the end of the exponential phase and from a corresponding biofilm obtained following a few hours of growth contain the same main groups of biomolecules: proteins (amide I, II, and III bands), nucleic acids (PO, signals near 1,240 and 1,080 cm^−1^), polysaccharides (PS, region from 1,150 to 900 cm^−1^), and lipids (signal near 2,900 cm^−1^) [7]. Biofilm growth can be monitored over time by following the evolution of the IR spectrum of the attached cells. As ATR/FT-IR allows the surface layers to be analyzed up to approximately 2 µm, it is well adapted to the study of the first stages of biofilm formation in situ under hydrated conditions [7],[8]. ATR/FT-IR can also be used to study dried, mature biofilms, as desiccation results in a significant decrease in biofilm thickness. In this case, a single biofilm cannot be monitored over time in a non-destructive manner, but samples can be taken at different times, dried, and analyzed [9]. The amide II band (N-H bending in proteins) is an efficient biomarker to monitor the quantitative sessile cells' evolution [5],[9]. Differences in some relative band intensities can be observed during biofilm growth, corresponding to changes of the amide II/PS and/or PO/PS band intensity ratio in the biofilm spectrum [7]–[9]. For example, the amide II/PS band intensity ratio evolution can reveal preferential extracellular polysaccharide production (ratio decrease), high accumulation of proteins (ratio increase), or preferential bacterial cells division (ratio stability) [7]–[9]. The PO/PS band intensity evolution can reveal preponderance of nucleic acids synthesis rather than polysaccharides during the first hours of bacterial adhesion, as shown for *P. fluorescens* [8]. Moreover, induction of biofilm detachment by starvation, revealed through the decrease of IR bands intensities characteristic of the biomass, can be observed after decreases in the dissolved organic matter concentration or after immersion of a biofilm in distilled water for several weeks [7],[10]. The evolution over time observed in the intensity of infrared bands, mainly corresponding to proteins, nucleic acids, and polysaccharides, allows for monitoring of biomass accumulation on a surface and for study of bacterial adhesion, biofilm growth, and detachment.

## 3. Sensitivity of biofilms to enzymatic treatments

When a chemical compound exhibits destabilization activity within a biofilm, it means that the molecules targeted by this compound are important for the integrity of the biofilm. Depending on the study, it is not always clear whether the compound has only the detachment-dispersion activity of the biofilm or if it also performs the destructive activity of the biofilm cells. The use of enzymes to treat attached microorganisms and biofilms was first applied in anti-fouling and therapeutic contexts [11],[12]. Thus, enzymes have been evaluated as cleaning agents and as agents for potentiating the action of antimicrobials. Proteolytic enzyme preparations alone or combined with ultrasonic waves remove a significant amount of *Escherichia coli* biofilm cells grown in milk on stainless steel [13]. Serine-proteases reduce the adhesion strength of spores and sporelings of the green alga *Ulva linza*, the diatom *Navicula perminuta*, and also inhibits settlement of cypris larvae of the barnacle *Balanus amphitrite* [12]. The anti-adherence effect of serine proteases is dependent upon the concentrations of specific enzymes. Serratiopeptidase is a metalloprotease produced by *Serratia*, with substrate specificity similar to that of the thermolysin produced by *Bacillus thermoproteolyticus* [14]. Serratiopeptidase enhances the activity of ofloxacin on sessile cultures of both pathogenic bacteria *Pseudomonas aeruginosa* and *Staphylococcus epidermidis* [11]. This activity enhancement depends upon enzymatic activity due to thermal inactivation of the protease, leading to absence of the effect. The presence of the enzyme at a concentration of 10 U/ml conduces a large decrease of sessile cells in the biofilm when used in conjunction with ofloxacin at a concentration equal to the minimal inhibitory concentration; an enzyme concentration of 10 U/ml with ofloxacin at a concentration equal to the minimal bactericidal concentration results in the absence of sessile cells. The presence of the enzyme alone conduces a slight decrease in biofilm formation of approximately one logarithmic unit. In this study, it has not been determined whether serratiopeptidase acts on EPS, on cellular proteins, or both.

Recent work showed that a 24 h protease treatment induce a breakdown of the *P. aeruginosa* and *Staphylococcus aureus* biofilm *in vitro* [15]. Among the enzymes tested, serine proteases were the most efficient enzymes against biofilms. Nevertheless, differences in efficiency were observed dependent upon the bacterial species and/or the enzyme used. Savinase had comparable high efficiency against both *P. aeruginosa* and *S. aureus* biofilms with a reduction of at least 70% of the sessile biomass. Subtilisin A was highly efficient against *S. aureus* biofilms, but was less efficient to detach *P. aeruginosa* cells. Immobilization of subtilisin A onto poly(ethylene-*alt*-maleic) anhydride copolymer films resulted in a decrease in *P. aeruginosa* adherence to the material without any effect onto *S. epidermidis* attachment [16]. This illustrates the involvement of surface proteins in *P. aeruginosa* and surface polysaccharides in the adherence of *S. epidermidis* to abiotic surfaces.

Proteases are not alone in their activity against biofilms. Alpha-amylase compounds derived from bacterial, fungal, and human origin also inhibit biofilm formation and detach biofilms of *S. aureus* in a concentration and time-dependent manner [17]. In a different study, alpha-amylase was shown to be more effective in detaching *P. aeruginosa* biofilm cells than *S. aureus* biofilm cells [15]. Alpha-amylase and serine proteases were the most effective enzymes for removal of biofilms of bacterial isolates from the food-industry [18]. Polysaccharidases were more efficient against *P. fluorescens* biofilms, while serine proteases were more efficient against *Bacillus* biofilms. The addition of surfactants and chelating agents to the enzyme solution enhanced the efficiency of polysaccharidases and proteases in removing biofilms [18]. The cation chelator ethylenediaminetetraacetic acid (EDTA) has been shown to induce *P. aeruginosa* biofilm dispersion and to exert bactericidal activity against both *P. aeruginosa* and *S. aureus* biofilm cells [19]. Moreover, EDTA can prevent the formation of and can be used in treating non-typeable *Haemophilus influenzae* (NTHI) biofilms [20]. The combination of EDTA and gentamicin, or EDTA, protease, and antiseptics results in the eradication of biofilms of various bacterial species [15],[19]. Cellulase treatment of the *P. aeruginosa* pellicle results in the dislocation of the structure into small fragments [21]. The presence of cellulase on poly(ethylene-alt-maleic) anhydride copolymer films induces a decrease in *S. epidermidis* adherence to the material without any effect onto *P. aeruginosa* attachment [16]. Again, this is an illustration of the involvement of polysaccharides in *S. epidermidis* adherence to abiotic surfaces.

Dispersin B (dspB) is a β-*N*-acetylglucosaminidase able to disperse and detach mature biofilms produced by *S. epidermidis* and other bacterial species which produce *N*-acetylglucosamine residues in β(1,6)-linkages [22]. The adsorption of dspB on different surfaces also resulted in a decrease of adherence of *S. epidermidis* to the material [23]. Moreover, mainly isolated adherent cells were observed after treatment and the combination of the enzyme treatment with an antibiotic induced a synergistic antimicrobial effect. Differences in the enzyme efficiency in the detachment of *Staphylococcus* biofilms have been observed as a function of variations in matrix constitution and between bacterial strains [24]. Thus, the efficiency of dspB is maximal with respect to the biofilms of the strains producing large quantities of poly-*N*-acetylglucosamine (PNAG), as these strains are not sensitive to proteases. Treatment with proteases has maximum efficacy towards strains producing little to no PNAG.

Before the study of Whitchurch and colleagues [25], DNA was thought to be a minor component of the biofilm structure. The addition of DNase I in growth medium was shown to prevent *P. aeruginosa* biofilm formation and the treatment of established *P. aeruginosa* biofilms resulted in the dissolution of 12, 36, and 60 h-aged biofilms with no significant effect on 84 h-biofilms. As shown with other enzymes, DNase I combined with EDTA increased the susceptibility of NTHI biofilms to ciprofloxacin and ampicillin [20]. eDNA is able to interact with multiple components involved in the formation of a biofilm: eDNA promotes bacterial adherence to hydrophobic surfaces, as well as cell aggregation through physicochemical interactions [26]–[28]; eDNA interacts with other EPS, including amyloids and exopolysaccharides [29]–[31]. Furthermore, eDNA acts as a nucleator of amyloid polymerization [29], it combines with the mannose rich exopolysaccharide Psl to form a network of fibres in the biofilm matrix of *P. aeruginosa* [30], and it binds to the glucose-rich cationic exopolysaccharide Pel in the biofilm stalk of *P. aeruginosa* [31]. Depending on the biofilm area, polysaccharide-eDNA interactions contribute to the structural stability of *P. aeruginosa* biofilms.

## 4. Extraction and purification of different types of EPS from the biofilm

EPS characterization through extraction is challenging due to the variety of EPS with different physicochemical properties and also because there is a need to detach EPS from microorganisms without destroying the cells. Thus, the characterization of the EPS components of a biofilm matrix relies on the combination of multiple extractions methods.

EPS extraction methods can be classified into two categories: physical methods and chemical methods. Physical extractions include ultrasonication, centrifugation, cation exchange resin, alternating current and heating, while chemical extractions include the use of alkaline, EDTA, and formaldehyde. Sugimoto et al. reported a simple method for extracting extracellular proteins from the biofilm matrix of *S. aureus* [32]. The principle of this method, based upon use of high concentrations of NaCl, is thought to be similar to ion exchange chromatography and may release EPS from cells. Many EPS molecules are positively or negatively charged and ionic interactions are essential to biofilm cohesion, in particular, EPS-cells interactions [33]. This type of extraction method, based on high NaCl concentrations, is highly efficient not only for matrix proteins but also for polysaccharides, eDNAs, and for different gram-positive and gram-negative bacteria [34]. Pan et al. conducted a comparative study of different EPS extraction methods from a natural algae-bacteria biofilm as follows [35]: EDTA (2%, 4 °C, 4 h), formaldehyde (36.5%, 4 °C, 1 h), formaldehyde (36.5%, 4 °C, 1 h), NaOH (1 M, 4 °C, 3 h), high-speed centrifugation (20,000 r/min, 4 °C, 20 min), ultrasonication (40 W in an ice bath, 2 min), and high-speed centrifugation (20,000 r/min, 4 °C, 20 min). The EPS extraction efficiency varied with the extraction method, with more EPS being extracted from chemical methods than from physical methods. Centrifugation was the least efficient technique, whereas the addition of EDTA was the most efficient extraction technique. Ultrasonication slightly increased the extraction efficiency of EPS. The addition of a NaOH treatment step to the use of formaldehyde considerably increased the extraction yield. Extraction with formaldehyde plus NaOH is efficient, likely due to induction of separation between acidic groups in EPS [36]. The EPS extraction yield can be improved by applying an alternating current after use of NaOH or EDTA [37]. The composition of the EPS extracts is affected by the extraction technique [35]: centrifugation extracts mainly polysaccharides; the addition of ultrasonication before centrifugation extracts high amounts of proteins and polysaccharides with the same efficiency as centrifugation alone; EDTA extracts high amounts of compounds with molecular weight less than 3,500 daltons (probably humic acid-like substances) and low amounts of polysaccharides and proteins; formaldehyde alone extracts mainly carbohydrates; the addition of NaOH after formaldehyde increases the protein content of the extract.

Some of these observations have been confirmed in other published studies on extracting EPS from different matrices. Formaldehyde with NaOH was shown to be effective in extracting EPS from aerobic activated sludge (AAS) [38]. Sonication and formaldehyde treatments were more efficient for extracting proteins, while EDTA was more efficient at extracting polysaccharides and humic acid substances from AAS [38],[39]. The addition of a precipitation step with 20% (w/v) trichloroacetic acid to EDTA treatment considerably enriched the EPS extract fraction in polysaccharides from biofilms [40].

The proteins of the matrix are diverse according to their content of ionic, hydrophobic, and neutral amino acids. Thus, Ras and collaborators developed a multi-method extraction protocol based on three types of extraction methods (mechanical, ionic, and hydrophobic) in order to obtain a high yield of diverse proteins from AAS [41]. This protocol consisted in discontinuous sonication (3 × 2 min), Tween (0.25%, 1 h), and EDTA (2% in Tris buffer, 1 h) treatments. The permutation of the EDTA and Tween steps had no effect on the total amount of extracted proteins. The successive application of the extraction protocol increased the protein extraction yield to 82–89% of the total soluble proteins harvested from AAS.

Applying a cation exchange resin (CER) for extraction of EPS is highly efficient for carbohydrates, proteins and DNA [42]. The principle of this method is based on the separation of the constituents of the biofilm that require cations for their cohesion. The extraction time and the CER dose are key factors to optimize the yield of EPS extraction [43]. The use of CER is particularly effective in separating EPS from cells. After extraction with CER, culturability of cells is not impaired [42]. The use of CER is a gentle extraction technique that does not alter the macromolecules and leaves no chemical residues that might interfere with the dosage or activity of extracted EPS [36]. The incubation of a biofilm suspension with CER followed by centrifugation is an efficient means of extracting matrix enzymes and measuring their activity [44]. Nevertheless, using CER is not always the most efficient way for extracting extracellular polymeric substances from sessile biomass [45].

One of the key factors for an efficient EPS extraction protocol is the limitation of cell lysis. There are different categories of approaches for quantifying cell lysis: measuring strictly intracellular enzyme activity, measuring cell-wall release, or quantifying the cells before and after extraction. The measurement of glucose-6-phosphate dehydrogenase (G6P-DH) activity is a good marker of the release of the intracellular content but is not applicable to certain EPS extraction techniques [41],[46]. Thus, an increase in pH through the use of NaOH denatures the enzymes and prevents measurement of their activity. EDTA, by chelating cations, can inhibit certain enzymatic activities. The damage to cell walls can be evaluated by 2-keto-3-deoxyoctonate (KDO) and N-acetylglucosamine (NAG) content changes in EPS extracts [47]. KDO and NAG measurements are useful when used in conjunction with the NaOH extraction process. Viability stains, known as the Live/Dead staining method, can be applied to detect and quantify cell lysis during EPS extraction [46],[47]; a combination of propidium iodide (PI) and FITC Annexin V or of PI and SYTO-9 followed by fluorescence microscopy or flow cytometry (FCM) measurement has been used with success [47],[48].

## 5. Purified EPS and artificial polymers to study the biofilm behavior

Biofilms are viscoelastic in nature and can be considered as polymer gels [2],[49]–[51]. Analysis of the mechanical behavior of separated matrix components and/or mixtures of such components may aid in understanding the role of EPS in both biofilm development and mechanical properties.

*E. coli* is the predominant aerobic gram-negative species of the normal intestinal microbiome of mammals but particular strains can be involved in intestinal or extraintestinal diseases. Colanic acid (CA) is a hydrophilic exopolysaccharide slime synthesized by mucoid strains of *E. coli* and other *Enterobacteriaceae* [52]. Colanic acid is a branched polysaccharide containing glucose, galactose, fucose, and glucuronic acid. Expression of CA by *E. coli* makes the cell surfaces hydrophilic and can reduce bacterial adherence to hydrophobic surfaces [53]. A mutant *E. coli* strain deficient in CA production has no altered adherence to polyvinylchloride, but has greatly affected biofilm development [54]. Moreover, the biofilms formed by the CA-deficient mutant are much less thick and less structured in space than the biofilms formed by the corresponding wild-type strain [54]. Bacterial cells initially adherent to abiotic surfaces are devoid of CA and express CA later during the development and maturation of the biofilm [55]. Indeed, CA synthesis is not observed in planktonic cells under normal laboratory growth conditions, but is induced inside biofilms [56]. Colanic acid expression is associated with colony mucoidy, suggesting that CA has a high molecular weight and high viscosity. Steady shear experiments on diluted and semi-diluted solutions of CA showed that viscosity is not affected by shear rate at low concentrations. At higher concentrations, shearing acceleration is associated with a viscosity decrease indicative of rheofluidification [57]. Biofilms are also subject to shear thinning that may result from the polymeric composition of the matrix [2]. In dynamic measurements, 1.5, 0.75 and 0.42% w/w CA solutions have a liquid-like behavior at weak frequencies (<0.6 rad s^−1^) [56]. With increasing frequencies, the storage modulus G′ increases more quickly than the loss modulus G″, revealing a viscoelastic fluid behavior. During oscillation analysis of a biofilm, the values for storage modulus are greater than the values for loss modulus in a large range of frequencies, showing that the matrix polymer content is concentrated enough to obtain viscoelastic behavior [2].

**Figure 1.**
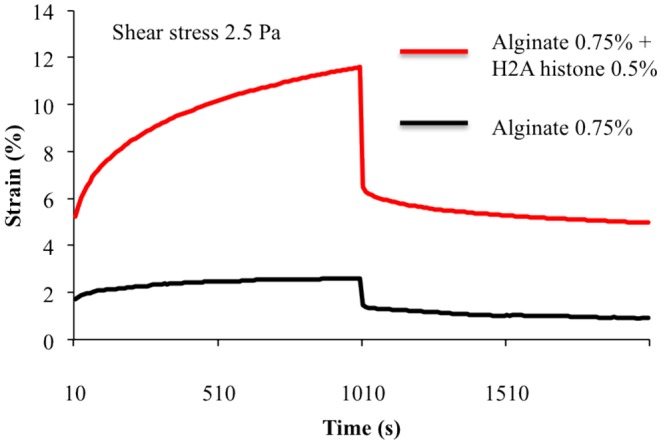
Creep test analysis showing the deformation and relaxation under stress of artificial matrices made of alginate and calcium or alginate, calcium, and H2A histone [61]. Percentages indicate the alginate and H2A histone contents of the matrix (weight/weight).

*P. aeruginosa* is an environmental species that causes opportunistic infections in patients with immunosuppression, burns, or cystic fibrosis. Mucoid strains of *P. aeruginosa* are mainly associated with chronic lung infections in patients with cystic fibrosis. A rheological analysis of an undiluted biofilm from a mucoid *P. aeruginosa* strain revealed that storage modulus G′, but not loss modulus G″, is dependent on temperature [58]. At temperatures between 20 and 50 °C, G′ values are greater than G″ values, indicating the presence of a viscoelastic solid. When temperature increases, only G′ decreases significantly, showing that the biofilm behaves increasingly as a viscous liquid. EPS extracted from this *P. aeruginosa* biofilm through centrifugation and filtration primarily contained extracellular polysaccharides with small amounts of proteins [58]. This EPS preparation showed shear-thinning behavior and revealed apparent viscosity dependence on the ionic strength of low molecular weight electrolytes, such as NaCl and NaBr. The typical polyelectrolyte polyacrylic acid, but not the polymer non-electrolyte polyvinyl alcohol, had the same behavior as EPS from *P. aeruginosa*, indicating the presence of similar molecular interactions within the two systems. Thus, carboxyl (or carboxylate) groups likely have a role in molecular interactions in EPS of *P. aeruginosa*. Structural changes in charged polymers, which occur due to the influence of an electrolyte, could be summarized as follows: initially, without additional ions, the repulsive Coulomb forces between anionic carboxyl groups induce the elongation of polymer chains. When electrolyte concentration increases, the cations partially compensate the charges. In these conditions, there is a gradual reduction in the repulsive forces, allowing the chain to adopt a random coil structure. At higher ion concentrations, molecular mobility decreases due to increasing particle agglomeration of the polymer, resulting in growing particle size [58]. The addition of a chaotropic substance, guanidine hydrochloride, had similar effects on the apparent viscosities of solutions of EPS, polyacrylic acid, and polyvinyl alcohol [58]. The apparent viscosities of the three compounds decrease at low concentrations of guanidine hydrochloride and then decrease more slowly at higher concentrations. Since guanidine hydrochloride increases the ability of soluble polymers to form intra- and intermolecular hydrogen bonds within or among chain molecules, EPS, polyacrylic acid, and polyvinyl alcohol are thought to have comparable hydrogen bonding characteristics. Polyvinyl alcohol and polyacrylic acid can form gel-like structures, mainly through the formation of hydrogen bonds. Thus, hydrogen bonds must be an essential binding force within the EPS network of the *P. aeruginosa* biofilm. Alginate is a major exopolysaccharide secreted by mucoid *P. aeruginosa* strains. This high molecular weight, negatively charged, and acetylated polymer is formed from β-1,4 linked D-mannuronic and L-guluronic acids [59],[60]. After secretion, alginate is not covalently linked to the cell surface, however, divalent cations can interact with alginate through ionic bonds, which leads to the formation of a gel. An alginate-calcium gel has a viscoelastic behavior similar to that of a *P. aeruginosa* biofilm, but exhibits much less stress under strain [61]. The addition of the cationic histone protein H2A to calcium and alginate leads to the formation of a gel with decreased storage modulus G′ and loss modulus G″, but increased stress under strain (Figure 1). H2A is hypothesised to be inserted in the narrow mesh formed by the interaction between calcium and alginate, form ionic bonds with alginate, and a create a wider mesh in some places, leading to increased deformability of the structure.

Non-mucoid strains of *P. aeruginosa* do not produce alginate but can produce Psl and Pel, two other extracellular polysaccharides [62]. Psl is a neutral, branched polysaccharide with a five-sugar repeat unit composed of D-mannose, D-glucose, and L-rhamnose. Acting as a promoter of bacteria-bacteria and bacteria-surface interactions during bacterial colonisation, Psl forms a fiber-like network within the matrix [63]. Pel is a cationic, *N*-acetylglucosamine and *N*-acetylgalactosamine rich polysaccharide [31]. Pel is involved in the initiation and maintenance of bacteria-bacteria interactions in biofilms [64]. Both Psl and Pel can be co-localized with eDNA in *P. aeruginosa* biofilms [30],[31]. Moreover, Psl can physically interact with DNA from different sources (*P. aeruginosa*, other bacterial species, human cells) to form fibers, which are further protected against enzymes targeting biofilm matrix components [30]. Hydrogen bonding is thought to mediate Psl-DNA interactions. eDNA interaction with heterologous matrix constituents can be modulated to control the viscoelastic behavior of the biofilm under mechanical stress [65]. When the alginate or Pel biofilm content increases, the yield strain of the biofilm under mechanical stress also increases [66]. When, Psl production increases, the elastic modulus of the biofilm also increases if the protein CdrA is co-produced with Psl. CdrA binds to mannose groups on Psl and acts as cross-linker between Psl molecules, reinforcing the elastic network in the biofilm matrix [66].

Extracellular proteins are important component of the matrix and amyloids are the major proteinaceous component of many biofilms [67]. Amyloids are non-covalently bound insoluble aggregates of proteins that form a fibrous cross-beta structure. Curli are filamentous structures expressed by *E. coli*, involved in bacterial adhesion to abiotic and biological surfaces and in biofilm formation [68],[69]. The major curli subunit CsgA harbors five successive repeating units involved both in the self-assembly of the protein and in interactions with fibronectin and other human proteins [69]–[71]. Peptides corresponding to the entire or to a portion of the repeat sequence R5 from CsgA form amyloid fibers *in vitro* [69],[71]. Cellulose is another crucial component of the extracellular matrix of *E. coli* biofilms [72]. Cellulose and curli over-expression induce large increases in bacterial adherence and biofilm formation [73]. Model matrices containing a mixture of methylcellulose and an amyloid peptide derived from the repeat sequence R5 of CsgA have increased deformability and instant elasticity when compared to methylcellulose alone [74]. The incorporation of different types of amyloid fibers in the polysaccharide matrix causes various responses to shear depending on the hydrophobicity of the auto-assembled peptide present and the morphology of corresponding fibers, such as flexibility or formation of large agglomerated structures [74]. Cellulose production during biofilm formation is observed in many bacteria [75]. The soil-dwelling, gram-positive bacterium *Streptomyces coelicolor* produces amyloidal fimbriae, which are anchored to the cell wall via cellulose and are involved in bacterial attachment to surfaces [76]. Thus, interactions between polysaccharides and amyloids are widely utilized by bacteria for adherence to surfaces and biofilm development.

*S. aureus* is a gram-positive pathogenic species that causes persistent biofilm infections [77]. *S. aureus* produces two types of amyloid structures: amyloid assembly of fragments from the biofilm-associated protein (Bap) occurring in response to environmental signals and amyloid assemblages composed of phenol soluble modulins (PSMs) [78]–[81]. *S. aureus* produces eDNA inside biofilms through the autolysis of a subpopulation of sessile cells [82]. After release in the biofilm matrix, eDNA interacts with PSMs to promote the formation of amyloid fibers [83]. Bap is involved in bacteria-bacteria interactions, bacterial adherence to surfaces, and biofilm formation [84]. Additionally, Bap is processed after bacterial autolysis and Bap fragments induce bacterial aggregation and biofilm formation [80]. Autolysis appears to induce biofilm formation in *S. aureus* through different EPS, i.e., eDNA, PSMs, and Bap.

In addition to molecular self-assembly between EPS in the matrix, colloidal self-assembly also occurs in biofilms. Colloidal self-assembly corresponds to the combination of bacterial cells with polysaccharide, protein, and eDNA components of EPS, leading to the formation of a viscoelastic material. These colloidal interactions result from physical forces between suspended particles, such as bacterial cells, and extracellular polymeric structures, influencing biofilm morphology and rheology [85]. Artificial staphylococcal biofilms composed of bacteria and chitosan mimic the structure and microrheology of natural biofilms under pH environments that induce an unstable matrix phase. Under these conditions, the molecular interactions between polymers creates matrix phase instability, allowing for colloidal self-assembly of the cells and polymers into a viscoelastic structure analogous to natural biofilms [85].

## 6. Conclusions

A biofilm is a heterogeneous and dynamic environment organized to optimize microbial functions. It consists mainly of water, cells, and microbial macromolecules. The content of the biofilm in cells and EPS strongly influences its structure and properties. Characterization of the biofilm matrix is a key element in understanding the biofilm phenotype. This includes determining the EPS content of the biofilm, studying interactions between EPS, and investigating the interactions between EPS and microbial cells. This characterization can be based on analytical chemistry techniques, on the use of enzymes to destabilize the structure, and on fractionation and purification approaches. ATR/FT-IR is well adapted to the study of attached cells and young biofilms in situ under hydrated conditions and can also be used to study dried mature biofilms. Proteases, polysaccharidases, and DNases can inhibit biofilm formation and detach biofilms with various efficiencies, depending on bacterial species, bacterial strains, and biofilm age. Protocols based on a combination of chemical (NaCl, EDTA, NaOH, detergent) and physical (sonication, centrifugation, cation exchange resin) treatments must be adapted to each particular biofilm to obtain optimum extraction efficiency for polysaccharides, proteins, and/or nucleic acids, while also limiting cell lysis. By making it possible to reconstitute artificial matrices and biofilms *in vitro*, the simplification and control of the composition of biofilm structure through the use of purified or artificial polymers and cells is a valuable tool for inferring the interactions between the various constituents and their roles in the organization and properties of biofilms. We still have much to learn about biofilms and a much progress to make to in order to master them.
